# Ginsenoside Rg1 Alleviates Acute Ulcerative Colitis by Modulating Gut Microbiota and Microbial Tryptophan Metabolism

**DOI:** 10.3389/fimmu.2022.817600

**Published:** 2022-05-17

**Authors:** Hao Cheng, Juan Liu, Dandan Zhang, Jing Wang, Yuzhu Tan, Wuwen Feng, Cheng Peng

**Affiliations:** ^1^ State Key Laboratory of Southwestern Chinese Medicine Resources, School of Pharmacy, Chengdu University of Traditional Chinese Medicine, Chengdu, China; ^2^ The Ministry of Education Key Laboratory of Standardization of Chinese Herbal Medicine, School of Pharmacy, Chengdu University of Traditional Chinese Medicine, Chengdu, China

**Keywords:** ginsenoside Rg1, gut microbiota, tryptophan, metabolomics, ulcerative colitis

## Abstract

Ulcerative colitis (UC) is a chronic and recurrent inflammatory disorder in the gastrointestinal tract. Here, we examined the pharmacological effects of ginsenoside Rg1, a natural compound with low bioavailability, on the acute experimental colitis mice induced by dextran sulfate sodium (DSS) and explored underlying mechanisms. Acute UC was induced in C57BL/6 mice by 2.5% DSS for 7 days, meanwhile, 2 mg/10 g b.w. ginsenoside Rg1 was administrated to treat the mice. Body weight, colon length, colon tissue pathology, and colon tissue inflammatory cytokines were assessed. The composition structure of gut microbiota was profiled using 16s rRNA sequencing. Global metabolomic profiling of the feces was performed, and tryptophan and its metabolites in the serum were detected. The results showed that Rg1 significantly ameliorated DSS-induced colonic injury and colonic inflammation. In addition, Rg1 also partly reversed the imbalance of gut microbiota composition caused by DSS. Rg1 intervention can regulate various metabolic pathways of gut microbiota such as valine, leucine, and isoleucine biosynthesis and vitamin B6 metabolism and the most prominent metabolic alteration was tryptophan metabolism. DSS decreased the levels of tryptophan metabolites in the serum, including indole-3-carboxaldehyde, indole-3-lactic acid, 3-indolepropionic acid, and niacinamide and Rg1 can increase the levels of these metabolites. In conclusion, the study discovered that Rg1 can protect the intestinal barrier and alleviate colon inflammation in UC mice, and the underlying mechanism is closely related to the regulation of gut microbiota composition and microbial tryptophan metabolism.

## Introduction

Ulcerative colitis (UC) is a chronic and idiopathic inflammatory disease that is characterized by relapsing and remitting mucosal inflammation, starting in the rectum and extending to proximal segments of the colon (1, 2). Clinical symptoms of UC mainly are urgency, incontinence, fatigue, increased frequency of bowel movements, mucus discharge, nocturnal defecations, abdominal discomfort, and bloody diarrhea (3). Epidemiological statistics demonstrated that the highest prevalence rates of UC in Europe (505 per 100,000), Canada (248 per 100,000), and the USA (214 per 100,000), and a gradual increase in the prevalence rates of UC in other developing countries or regions such as Asia, the Middle East, and South America (3). In addition, many UC patients require surgery and are still difficult to cure after surgery, which brings a serious financial burden on the families of UC patients. Therefore, UC is listed as a modern refractory disease by the World Health Organization (4). The current treatment methods for UC are mainly anti-inflammatory and immunosuppressive agents, such as amino salicylates, corticosteroids, and immunosuppressive medications (2, 3). However, these methods often are accompanied by various adverse effects, such as infection, fever, diarrhea, and high recurrence rates (5). Therefore, finding a new safe, affordable, and effective therapeutic method for UC is urgent.

The etiology of UC is complex, among which genetic susceptibility, immune response, and the imbalance of gut microbiota have been considered the main key contributing factors (6). Gut microbiota plays an indispensable role in various physiological functions, such as energy metabolism (7), digestion and absorption of nutrients (8), and host immune homeostasis (9). The imbalance of gut microbiota has been shown to be associated with the development of UC and the regulation of gut microbiota by drugs and administration of beneficial bacteria can effectively alleviate intestinal inflammation (10). In addition to gut microbiota composition, gut microbiota metabolites, including bile acids, short-chain fatty acids, and tryptophan (Trp), are also playing an important role in maintaining the integrity of the intestinal barrier and immune homeostasis (11). Dietary-derived Trp can be bio-transformed by the gut microbiota into a series of microbial metabolites, including indole propionic acid (IPA), indole acetic acid (IAA), and 3-indolylacrylic acid (ILA). These microbial metabolites of Trp have been confirmed as the ligands of the aryl hydrocarbon receptor (AHR) which are significant for intestinal immune homeostasis and intestinal barrier function (12). Dietary-derived microbial metabolites of Trp can increase the intestinal epithelial tight junction protein by blinding AHR (13). In addition, compared with normal mice, intestinal epithelium-specific AHR deficiency mice are more sensitive to DSS-induced intestinal inflammation and had enhanced loss of epithelial cells (14). AHR also plays an important role in the physiological activity of various intestinal immune cells, such as innate lymphoid cells, myeloid cells, and Th17/Th22 cells (15). These studies suggest the importance of gut microbiota and microbial Trp metabolites on intestinal immune homeostasis and intestinal disease including UC.

**Figure 1:**
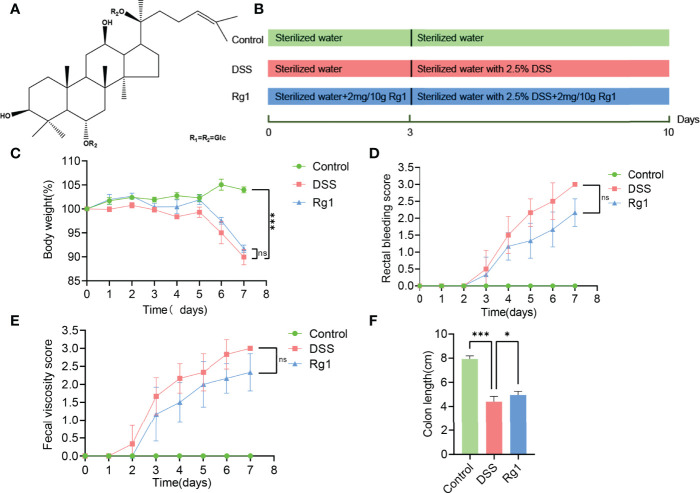
The effect of Rg1 on the DSS-induced UC mice. **(A)** Chemical structure of ginsenoside Rg1. **(B)** Inducement of UC mice model and the treatment of Rg1. **(C)** Daily changes in body weight of each group during UC mice model induction. **(D)** Changes in fecal bleeding of each group during UC mice model induction. **(E)** Changes in fecal viscosity of each group during UC mice model induction. **(F)** Colon length was measured for each group on the 7^th^ day. Significance levels are indicated as *P < 0.05, ***P < 0.001 and ns stands for not statistically significant.

*Panax ginseng C. A.* Meyer, also known as “ginseng,” is a tonic widely used in many countries (16). Ginsenoside Rg1 (C_42_H_72_O_14_, Rg1, **Figure 1A**) is one of the bioactive constituents of ginseng (17). Rg1 has strong pharmacological activity and has been discovered to have a beneficial effect on the treatment of various diseases, such as obesity (18), type 2 diabetes (19), nonalcoholic fatty liver (20), cancer (21), hyperlipidemia (22), acute lung injury (23), nephritis (24), and arthritis (25). A wide range of studies has disclosed the pharmacological effect of Rg1 on UC (17, 26). However, given the low oral bioavailability (the area of plasma concentration-time curve after 200 mg/kg Rg1 oral administration was 2.81 ± 1.13 μg/ml per h in the rats) of Rg1 (27), it cannot fully explain the pharmacological mechanism of Rg1 from the perspective of oral bioavailability. Recently, more and more studies demonstrated that drugs with low oral bioavailability can interact with the gut microbiota to exert pharmacological activity, for example, berberine (28). The regulation of Rg1 on gut microbiota has also been reported in many pieces of research. The pharmacological effect of Rg1 has been suggested to be correlated to the regulation of Rg1 on gut microbiota (29–31). Based on the above facts, we hypothesize that gut microbiota and microbial metabolites of Trp may be the key point to disclosing the pharmacological mechanism of Rg1 on UC.

To verify our conjecture, in this study, we examined the protective effects of Rg1 on DSS-induced UC mice and explored the underlying mechanism from the perspective of gut microbiota composition and gut microbiota metabolism for the first time. Phenotypic indicators, including body weight, colon tissue pathological changes, and colon tissue inflammatory cytokine of UC mice were detected and analyzed to evaluate the anti-colitis activity of Rg1. The gut microbiota of UC mice was profiled by sequencing the bacterial 16S rRNA gene V3-V4 region. In addition, the metabolic changes in feces were determined by ultra-performance liquid chromatography coupled with mass spectrometry (UPLC-MS). The levels of Trp metabolites in the serum were performed to comprehensively evaluate the effects of DSS and Rg1 on the Trp metabolism.

## Materials and Methods

### Materials

Ginsenoside Rg1 (purity > 98%) was purchased from Herbpurify CO., LTD (Chengdu, Sichuan Province, People’s Republic of China). DSS (molecular weight: 36-50 kDa) was bought from MP Biomedicals Inc. (Aurora, OH, USA). MS-grade formic acid was purchased from TCI (Shanghai, China) and methanol was purchased from Thermo (USA). Ultra-pure water was produced by a Milli-Q water purification system (Millipore, MA, USA). Methanol (Purity > 99.0%) used was obtained from Thermo Fisher Scientific (USA). The reference substance of Trp and its metabolites used were as follows: Nicotinamide (Nam, Sinopharm Chemical Reagent Co., Ltd, China); Indole-3-formaldehyde (IAld, Yuanye, Shanghai, China); 3-Indolepropionic acid (IPA, Aladdin, Shanghai, China); Tryptophan (Trp, Sinopharm Chemical Reagent Co., Ltd, China); and 3-Indolyllactic acid (ILA, Aladdin, Shanghai, China).

### Establishment and Treatment of UC Animal Model

Eighteen SPF male C57BL/6 mice with a body weight of 20 ± 2g were purchased from Chengdu Dossy Experimental Animal Co., LTD., (NO. SCXK 2020-030, Chengdu, China). All experiments involving animals were agreed upon by the Animal Ethics Committee at Chengdu University of Traditional Chinese Medicine. Animals were adapted to the SPF environment with a relative humidity of 60 ± 5%, a standard 12h light/dark cycle, and a temperature of 22 ± 2°C. The animals were allowed free access to standard chow and sterilized water. After 5 days of acclimatization, the mice were randomly divided into three groups (n = 6 per group) (1): the Control group was allowed free access to standard chow and sterilized water for 10 days (2); the DSS group was allowed free access to standard chow and sterilized water in the first three days and drinking water was changed to sterile water with 2.5% DSS (w/v, dissolved in sterilized water) on the 4^th^ day for 7 days; and (3) the Rg1 group was allowed free access to standard chow and sterilized water in the first 3 days and drinking water was changed to sterile water with 2.5% DSS on the 4^th^ day for 7 days, in addition, the mice were administered Rg1 daily for 10 days in the dosage of 2mg/10g b.w (17, 26). **Figure 1B** shows the detailed grouping and treating of mice.

### Assessment of Daily Disease Activity and Sample Collection

Throughout the experiment, the body weight, stool consistency, and rectal bleeding of mice in each group were recorded daily. The scoring of stool consistency and rectal bleeding has been described elsewhere (32). Briefly, stool consistency was scored as follows: 0, normal; 1, soft but still formed; 2, very soft; 3, diarrhea. Rectal bleeding was scored as follows: 0, hematochezia positive; 1, hematochezia negative; 2, blood traces in stool visible; 3, rectal bleeding. The feces of mice on the 6^th^ and 7^th^ days were taken for detection of gut microbiota composition and fecal sample metabolites, respectively. All collected feces from mice were stored at -80°C. On the 10^th^ day, mice serum was obtained by enucleation and was drawn into a heparinized EP tube, and then centrifuged at 3000 r for 10 min in 4°C. The serum was then transferred into new EP tubes and stored at -80°C before analysis of serum metabolites. Then, the mice were dissected, and the complete colon was taken and measured. The obtained colon was divided into two sections, the section of the colon tissue used for pathological examination was fixed with 4% paraformaldehyde, and another section of colon tissue used for inflammatory cytokine detection was stored at -80°C.

### Histopathological Examination

The colon tissues fixed in 4% paraformaldehyde were embedded in paraffin. The colon tissues were then cut at 4 μm thickness and two pieces were taken, followed by hematoxylin and eosin (H&E) staining and alcian blue-periodic acid sthiff (AB-PAS) staining, respectively. In addition, the pathological changes of colon tissue were scored according to the following rules: inflammatory cell infiltration (0–3), crypt distortion (0–3), and colon mucous membrane detachment (0–3) (33).

### Immune Inflammatory Factor Detection

The colon tissue stored at -80°C was weighed and mixed with phosphate-buffered saline at a weight (mg) to volume (μl) ratio of 1:9. After that, the colon tissue was ground with a tissue grinder. The prepared tissue homogenate was centrifuged at 3000 r for 5 min at 4°C, and the supernatant was taken for the detection of inflammatory cytokine in colon tissue. The protein levels of IL-2 and TNF-*α* were measured by the BD Cytometric Bead Array Mouse Th1/Th2/Th17 Cytokine Kit (BD Biosciences, USA) according to the manufacturer’s instructions. Finally, the obtained streaming data were processed by FCAP v3.0 software, and the concentration of cytokines was calculated according to the standard curve.

### Analysis of Gut Microbiota Composition

The DNA of gut microbiota was extracted by using the E. Z. N. A. ^®^ soil DNA Kit (Omega Bio-Tek, Norcross, GA, U.S.). The primers used for bacterial 16S rRNA gene amplification were as follows: the forward primer pair was 338 F: 5’-ACTCCTACGGGAGGCAGCAG-3’ and the reverse primer was 806 R: 5’-GGACTACHVGGGTWTCTAAT-3’. The instrument was used to amplify the hypervariable region V3-V4 of bacterial 16S rRNA gene is an ABI GeneAmp^®^ 9700 PCR thermocycler (ABI, CA, USA). The amplification and sequencing steps were roughly divided into three steps. First of all, the PCR amplification of the 16S rRNA gene was performed as follows: initial denaturation at 95°C for 3 min, followed by 27 cycles of denaturing at 95°C for 30 s, annealing at 55°C for 30 s, and extension at 72°C for 45 s, and single extension at 72°C for 10 min, and end at 4°C. Subsequently, the PCR product was extracted from 2% agarose gel and purified by using the AxyPrep DNA Gel Extraction Kit (Axygen Biosciences, Union City, CA, USA) according to the manufacturer’s instructions and quantified using Quantus™ Fluorometer (Promega, USA). Finally, purified amplicons were pooled in equimolar and paired-end sequenced on an Illumina MiSeq PE300 platform/NovaSeq PE250 platform (Illumina, San Diego, USA) according to the standard protocols by Majorbio Bio-Pharm Technology Co. Ltd. (Shanghai, China).

The raw 16S rRNA gene sequencing reads obtained were demultiplexed and quality-filtered by FASTP (version 0.20.0). Subsequently, the raw 16S rRNA gene sequencing reads were merged by FLASH (version 1.2.7) with the following three conditions: (1) the 300 bp reads were truncated at any site receiving an average quality score of < 20 over a 50 bp sliding window, and the truncated reads shorter than 50 bp were discarded, reads containing ambiguous characters were also discarded; (2) only overlapping sequences longer than 10 bp were assembled according to their overlapped sequence. The maximum mismatch ratio of the overlap region is 0.2. Reads that could not be assembled were discarded; and (3) Samples were distinguished according to the barcode and primers, and the sequence direction was adjusted for exact barcode matching, and 2 nucleotide mismatches in primer matching. Operational taxonomic units (OTUs) with a 97% similarity cutoff were clustered using UPARSE (version 7.1), and chimeric sequences were identified and removed. The taxonomy of each OTU representative sequence was analyzed by RDP Classifier (version 2.2) against the 16S rRNA database (eg. Silva v138) using a confidence threshold of 0.7.

### Handling of Fecal Samples for Metabolic Profiling

Fecal samples of about 62.1-100.9 mg ( ± 1%) were transferred into a 2 ml EP tube and then mixed with 0.6 ml 2-chlorophenyl alanine (4 ppm) methanol (-20°C).After vortex mixing for 30 s, ground at 60 Hz for 90 s, and then an ultrasound at room temperature for 10 min. After sonication, centrifuged at 4°C and 12000 r for 10min, taken 300 μl supernatant, filtered through 0.22 μm membrane, and put the filtrate in the test bottle. Twenty μl of each sample was mixed into a QC sample to correct the deviation of the analysis result of the mixed sample and the error caused by the analysis instrument itself. The remaining test samples were analyzed by UPLC-MS global profiling of feces metabolites technology.

### UPLC-MS/MS Global Metabolites Profiling, Data Processing, and Metabolites Identification of Feces Samples

In the same chromatographic condition and mass spectrometry condition, UPLC-MS/MS was adopted to analyze the metabolites of feces samples. Chromatographic separation was used with Vanquish UHPLC (Thermo Fisher Scientific, USA) and an ACQUITY UPLC^®^ HSS T3 (150×2.1 mm, 1.8 µm, Waters) column maintained at 40°C. The temperature of the autosampler was 8°C. The mobile phase in positive ion mode was 0.1% formic acid water (C)-0.1% formic acid acetonitrile (D) or in negative ion mode was 5 mM ammonium formate water (A)-acetonitrile (B) at a flow rate of 0.25 ml/min. Injection of 2 μl of each sample was done after equilibration. The gradient elution conditions were as follows: 0~1 min, 2% B/D; 1~9 min, 2%~50% B/D; 9~12 min, 50%~98% B/D; 12~13.5 min, 98% B/D; 13.5~14 min, 98%~2% B/D; 14~20 min, 2% D-positive model (14~17 min, 2% B-negative model). The ESI-MSn experiments were used with the spray voltage of 3.5 kV and -2.5 kV in positive and negative modes, respectively. Sheath gas and auxiliary gas were set at 30 and 10 arbitrary units, respectively. The capillary temperature was 325°C. The orbitrap analyzer was scanned over a mass range of 81-1000 m/z for a full scan at a mass resolution of 70000. Data-dependent acquisition MS/MS experiments were performed with an HCD scan. The normalized collision energy was 30 eV. Dynamic exclusion was implemented to remove some unnecessary information in MS/MS spectral.

Before performing further multivariate comparisons, quality assurance (QA) inspections were performed on the obtained mass spectrometry data to ensure the reliability of the data quality. Principal component analysis (PCA) was adopted to show the aggregation and dispersion of all samples. Orthogonal projections to latent structures discriminant analysis (OPLS-DA) were used to find potential differential metabolites. Variables with variable importance for projection (VIP ≥ 1.0 and *P* < 0.05) in the OPLS-DA model were preserved. Significant different variables between two groups were determined using one-way analysis of variance (ANOVA) (*P* < 0.05), and only variables showing a dose-effect relationship were preserved and regarded as differential metabolites. After confirming the accurate molecular weight of the metabolites (molecular weight error < 30 ppm), the fragment information obtained in the MS/MS mode was further matched and annotated with each database to determine the accurate metabolite information. The online databases include HMDB (www.hmdb.ca), Metlin (metlin.scripps.edu), massbank (www.massbank.jp), LipidMaps (www.lipidmaps.org), and mzclound (www.mzcloud.org). Non-target metabolic analysis, including QA, PCA, OPLA-SD, and volcano maps were all performed on the metabolomics cloud platform (www.biodeep.cn).

### Quantitative Analysis of Tryptophan and Its Metabolites in Serum

Weighed an appropriate amount of Trp and its metabolite standards and prepared a single-label mother liquor with 50% methanol. Prepared a mixed standard product from each mother liquor, diluted one by one to an appropriate concentration with 10% methanol to make a working standard solution. The mother liquor and working standard solution were stored at 0°C. In addition, the concentration of Trp and its metabolites in the standard is shown in **Table S1**. Took an appropriate amount of serum sample into a 2 ml EP tube and mixed with 100 μl of 80% methanol aqueous solution and 900 μl of 10% methanol aqueous solution successively. Then centrifuged at 12000 r and 4°C for 5 min. One hundred μl of the internal standard solution with a concentration of 10 ppb was mixed with 100 μl of the supernatant and then filtered with a 0.22 μm filter membrane after vortex for 30 s. The filtrate was stored in the detection bottle for subsequent HPLC-MS/MS analysis.

With the same chromatographic condition and mass spectrometry condition, the levels of Trp and its metabolites in the serum were analyzed by HPLC-MS/MS. HPLC (Waters, USA) and ACQUITY UPLC^®^ HSS T3 column (2.1×150 mm, 1.8 μm) (Waters, USA) were used for chromatographic separation. Passed each 5 ul standard solution and test solution through the column at a flow rate of 0.3 ml/min and the column temperature was 40°C. The mobile phase consists of 0.1% methanol-water (A) and 0.1% formic acid methanol (B). Performed gradient elution according to the following conditions: 0~2 min, 1%B; 2~3 min, 1~30% B; 3~3.5 min, 30% B; 4.5~8 min, 30~50% B; 8~10 min, 50 ~95% B; 10~11 min, 95% B; 11~17 min, 95~1% B. Mass spectrometer AB5000 (AB SCIEX) was used to identify the isolated Trp and its metabolites. The sample eluted from the column was ionized with an electrospray ionization source in the positive ionization mode. The ion source temperature was 500°C and the ion source voltage was 5500 V. The voltages of collision gas, curtain gas, atomizing gas, and auxiliary gas were 6, 30, 50, and 50 psi, respectively. Scanned and analyzed the samples and standards by using multiple reaction monitoring and locked retention time. The ion pairs used for quantitative analysis are shown in **Table S2**.

### Statistical Analysis

All data are presented as mean ± standard deviation (SD) except for the Alpha diversity indexes (Ace, Chao, and Shannon). The significant difference was evaluated by using ANOVA when groups were more than two (nonparametric Kruskal-Wallis rank-sum test was used when one-way ANOVA analysis was not suitable). In addition, Dunnett’s T3 multiple comparisons test was adopted to evaluate the significant difference when the data are normally distributed but have unequal variances. *P* < 0.05 was considered to be statistically significant. Significance levels are indicated as *P < 0.05, **P < 0.01, and ***P < 0.001.

## Results

### Rg1 Can Protect the Intestinal Barrier and Alleviate Colon Inflammation in DSS Induced UC Mice

**Figure 2:**
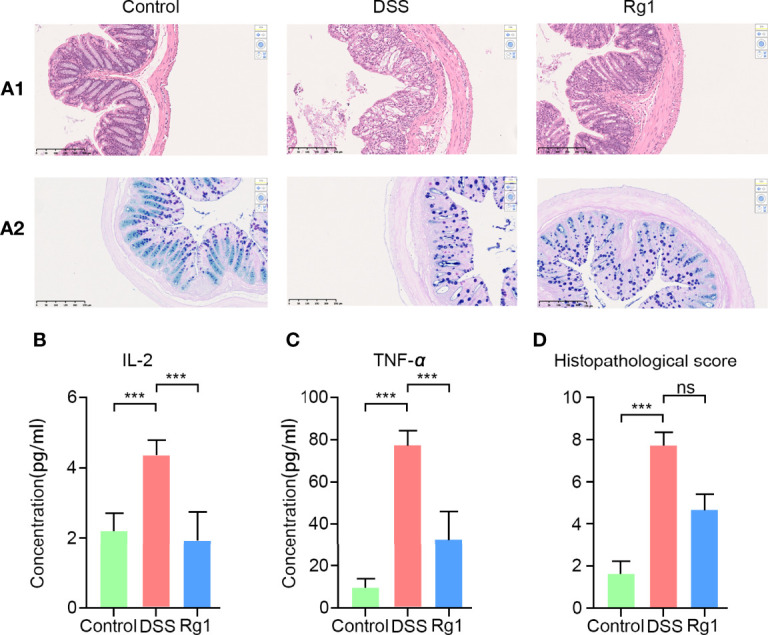
The changes of colonic histopathology and inflammatory factors. **(A-1)** HE staining results in colon tissue. **(A-2)** AB-APS staining results of colon tissue. **(B, C)** The levels change of inflammatory factors including in colon tissues of each group, including IL-2 and TNF-*α*. **(D)** Histopathological score of three groups. *P* < 0.05 was considered to be statistically significant. Significance levels are indicated as ***P < 0.001 and ns stands for not statistically significant.

The symptoms of DSS-induced acute ulcerative colitis are similar to human UC (2). As shown in **Figure 1**, we observed that compared with the control group, an oral challenge of 2.5% DSS can result in significant weight loss (*P* < 0.001, **Figure 1C**), fecal bleeding (P < 0.001, **Figure 1D**), and diarrhea (P < 0.001, **Figure 1E**), especially on the 7^th^ day, *P* < 0.001). However, no significant effect of Rg1 on DSS-induced UC symptoms was observed. In addition to observing the effect of Rg1 on clinical symptoms of UC mice, we examined the effects of Rg1 and DSS on colon tissue of UC mice. DSS can significantly shorten the colon compared with the control group (*P* < 0.001), however, the colon length of the Rg1 group was significantly longer compared with the DSS group (*P* < 0.05, **Figure 1F**). According to H&E staining results, the colon structure of the DSS group was severely damaged, mainly manifested as the destruction of the crypt structure, the disappearance of the glands, and the increase of inflammatory infiltration compared with the control group (**Figure 2A-1**). In addition, the goblet cells of the DSS group are greatly reduced compared with the control group. However, destruction of intestinal barrier structure caused by DSS was significantly alleviated after Rg1 treatment as shown in **Figure 2A-2**. Inflammation is one of the main pathological manifestations of UC and also is one of the important indicators for evaluating the severity of UC (34). Therefore, we detected the levels of inflammatory cytokines in colon tissue. As shown in (**Figures 2B, C** **)**, the levels of inflammatory cytokines including IL-2 (*P* < 0.001) and TNF-*α* (*P* < 0.001) in colon tissue were significantly increased in DSS groups compared with the control group. However, the abnormally increased level of IL-2 (*P* < 0.001) and TNF-*α* (*P* < 0.001) induced by DSS was reduced after Rg1 treatment. In addition, the histopathological scores also showed that the DSS challenge caused severe pathological changes in colon tissue, however, Rg1 had no significant effect on the histopathological scores of UC mice (**Figure 2D**).

### The Influence of Rg1 on Gut Microbiota Composition

**Figure 3:**
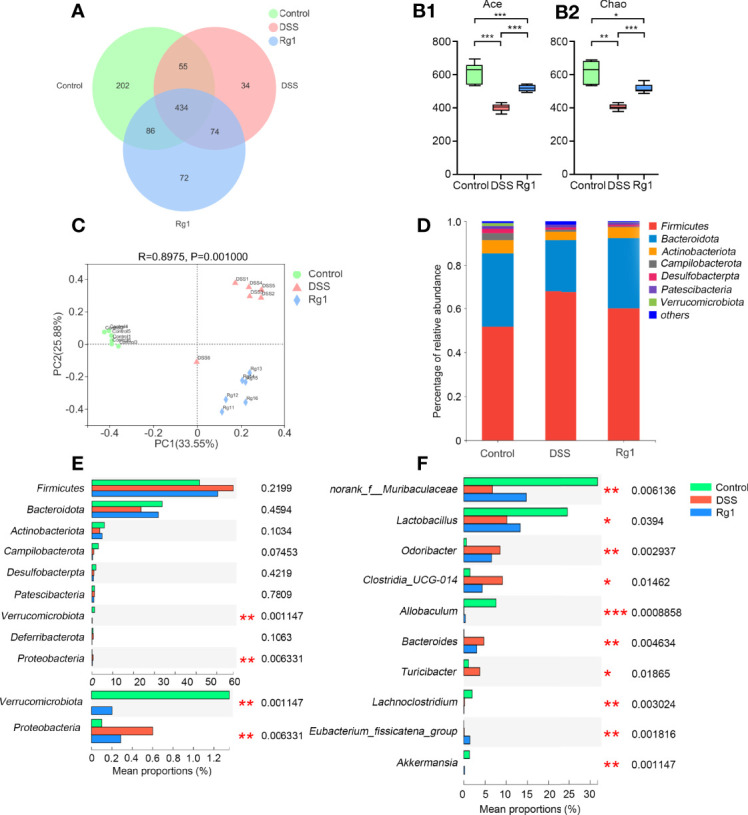
The change of the gut microbiota after DSS and Rg1 intervention. **(A)** The Venn diagram shows the overlap of OTUs in each group. Alpha diversity indicators: Ace **(B-1)** and Chao **(B-2)** show the change in community richness. The bars represent the maximum, upper quartile, median, lower quartile, and minimum from top to bottom. **(C)** PCoA analysis reflects the similarities and differences among the three groups. **(D)** Histogram shows the percent of community abundance on the phylum level. **(E)** The differential bacteria among three groups at the phylum level. **(F)** The differential bacteria among three groups at the genus level. *P* < 0.05 was considered to be statistically significant. Significance levels are indicated as *P < 0.05, **P < 0.01, and ***P < 0.001.

The imbalance of gut microbiota composition has been reported to be closely associated with the occurrence and development of UC (32, 35). Accordingly, we examined whether Rg1 and DSS can regulate the overall structure of gut microbiota. After OTU clustering, a dilution curve was constructed to evaluate the microbial diversity of each sample. It can be seen from the trend of the dilution curve that the dilution curve gradually becomes flat as the number of sequencing increases, indicating that the sample species diversity is high and the data are reliable (**Figure S1**). In addition, the Pan/Core graph reflects that as the sample size increases, the total OTUs number increases slowly, and the number of OTUs shared by each group tends to no longer decrease (**Figures S2A, S2B**). These results also demonstrate that the amount of test sample is sufficient. A total of 957 OTUs were annotated in all samples. 434 OTUs were present in all groups and 202, 34, and 72 OTUs were uniquely present in the control group, the DSS group, and the Rg1 group, respectively (**Figure 3A**). DSS can significantly decrease the community richness (Ace and Chao, **Figures 3B-1, B-2**) compared with the control, and Rg1 could significantly increase the community richness of gut microbiota compared with the DSS group. In addition, the community diversity of the DSS groups was dramatically lower (Shannon) compared with the control group (**Figure S2C**). Principal coordinate analysis (PCoA) of the bray-Curtis distance based on OTUs showed the changes in the overall structure of gut microbiota in mice after DSS treatment (**Figure 3C**). Significant changes in gut microbiota were observed along PC1 and PC2 in the DSS group and Rg1 group compared with the control group. In addition, the sample hierarchical clustering tree based on OTUs shows significant differences among the three groups, and the Rg1-treated samples are clustered separately from the DSS group (**Figure S2D**).

To identify changes in specific strains and search the differential bacteria which may be responsible for the occurrence and mitigation of colitis among three groups, the change in gut microbiota at the phylum level and the genus level is analyzed and is shown in **Figure 3**. At the phylum level, the detected bacteria (> 90%) in all samples belong to the following seven phyla: *Firmicutes*, *Bacteroidota*, *Actinobacteriota*, *Campilobacterota*, *Desulfobacterpta*, *Patescibacteria*, *and Verrucomicrobia* (**Figure 3D**). The relative abundance of *Verrucomicrobia* was significantly reduced and that of *Proteobacteria* was significantly increased in the DSS group compared with the control and Rg1 group (**Figure 3E**). At the genus level (**Figure 3F**), compared with the control group, the relative abundance of *norank_f_Muribaculaceae*, *Lactobacillus*, *Allobaculum*, and *Akkermansia* was significantly reduced and that of *Odoribacter*, *Clostridia_UCG-014*, *Bacteroides*, and *Turicibacter* was significantly increased in DSS group. After Rg1 treatment, *norank_f_Muribaculaceae*, *Lactobacillus*, *Allobaculum*, and *Akkermansia* were significantly enriched and *Odoribacter*, *Clostridia_UCG-014*, *Bacteroides*, and *Turicibacter* were significantly restrained compared with the DSS group.

### The Influence of Rg1 on Gut Microbiota Metabolism

**Figure 4:**
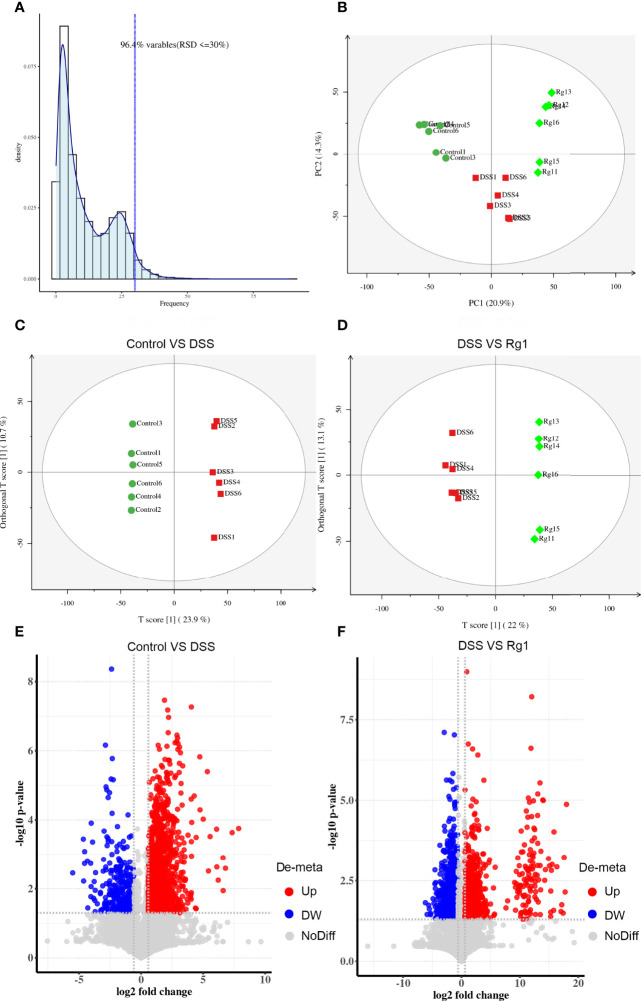
Rg1 modulated the fecal metabolism in ESI positive ion mode. **(A)** QA analysis result shows the reliability of the data. **(B)** PCA score plots show that reflect the distribution of all samples. **(C)** OPLS-DA sore plot reflects the difference between the Control group and the DSS group. **(D)** OPLS-DA sore plot reflects the difference between the DSS group and the Rg1 group. **(E)** The volcano map reflects the different specific metabolites between the Control group and the DSS group. **(F)** The volcano map reflects the different specific metabolites between the DSS group and the Rg1 group.

As a system biology method, metabolomics has been used as a powerful tool to study the relationship between gut microbiota metabolism and the host (36). Therefore, the change of metabolites in fecal samples after the DSS challenge and Rg1 treatment were analyzed by global UPLC-MS/MS profiling. **Figures 4**, **S3** show the influence of Rg1 on the gut microbiota metabolism in positive and negative modes, respectively. QA analysis of the raw data implied good data quality (**Figures 4A**, **S3A**). PCA score plot displayed a distinct clustering of fecal samples among three groups in both positive and negative modes (**Figures 4B**, **S3B**), indicating that DSS and Rg1 could influence gut microbiota metabolism. Then, the variables were screened that contribute to the separation of each group by OPLS-DA. The score plots in positive and negative modes showed that the DSS group can be clearly separated from the control group (**Figures 4C**, **S3C**), and the Rg1 group can be clearly separated from the DSS group (**Figures 4D**, **S3D**). Then, based on OPLS-DA, VIP ≥ 1 value and *P* < 0.05 value were used to screen potential variables responsible for group separation. The volcano plot reflected the differential variables and their direction of change between the compared group (**Figures 4E, F**, **S3E, F**).

**Figure 5:**
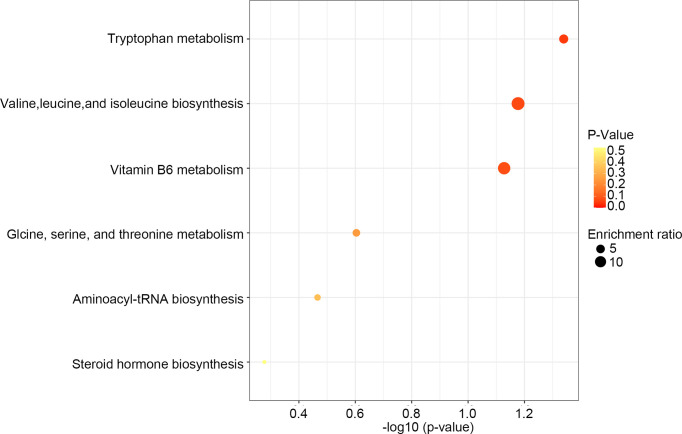
KEGG pathway enrichment analysis of differential metabolites.

After comparing the fragment information obtained in MS/MS mode with each database and further matching and annotation, the potential difference metabolites can be obtained through further analysis and screening. As shown in **Table 1**, the levels of 13 metabolites were significantly changed in the DSS group compared with those in the control group, but these changes were partly recovered after Rg1 treatment. These metabolites include *L*-threonine, 5-hydroxyindoleacetic acid, 4-pyridoxic acid, taurochenodesoxycholic acid, indole-3-acetaldehyde oxime, 1-aminocyclopropanecarboxylic acid, 4-pyridoxic acid, etc. Among the metabolism pathways related to these metabolites, bile acid metabolism, Trp metabolism, and vitamin metabolism may contribute to the treatment of UC. In addition, based on these 13 differential metabolites, enrichment analysis of the KEGG pathway of differential metabolites showed that the most prominent metabolic alteration was Trp metabolism (**Figure 5**).

**Table 1:** The differential metabolites with the dose-effect relationship among three groups.

Metabolites	Formula	KEGG ID	Control	DSS	Rg1	KEGG Pathway
*L*-Threonine	C_4_H_9_NO_3_	C00188	447705761.9 ± 99551783.5 (↑) *	259600031.2 ± 51278018.4	605292999.3 ± 150217581.4 (↑) ***	Microbial metabolism in diverse environments
5-Hydroxyindoleacetic acid	C_10_H_9_NO_3_	C05635	291228058.0 ± 68660808.5 (↑) **	153691174.6 ± 47275886.7	218342981.3 ± 3863 6244.2 (↑)	Trp metabolism
4-Pyridoxic acid	C_8_H_9_NO_4_	C00847	135833468.1 ± 9917234.6 (↑) *	104734226.2 ± 25477186.1	136917876.2 ± 13974922.3 (↑) *	Vitamin B6 metabolism, microbial metabolism in diverse environments
Indole-3-acetaldehyde oxime	C_10_H_10_N_2_O	C02937	60616181.2 ± 39953744.6 (↑)	14302151.9 ± 7952126.0	108929041.2 ± 63684430.5 (↑) *	Trp metabolism biosynthesis of secondary metabolites
1-Aminocyclopropanecarboxylic acid	C_4_H_7_NO_2_	C01234	241435732.7 ± 60728484.7 (↑) *	140687898.5 ± 32356677.4	295481992.3 ± 84151057.4 (↑) **	Cysteine and methionine metabolism, biosynthesis of secondary metabolites
9,10-Dihydroxyoctadec-12-enoic acid	C_18_H_34_O_4_	C14828	71376038.1 ± 15429421.0 (↑) *	35759747.1 ± 11275257.8	74733821.5 ± 27142255.3 (↑) *	Linoleic acid metabolism
Taurochenodesoxycholic acid	C_26_H_45_NO_6_S	C05465	4430335.1 ± 1849953.8 (↓) **	12583710.0 ± 4832203.9	5968637.2 ± 2835139.9 (↓) *	Primary bile acid biosynthesis, secondary bile acid biosynthesis
Diethylphosphoric acid	C_4_H_11_O_4_P	C06608	19487239.5 ± 757738.7 (↓) **	22200276.7 ± 1640303.2	14557855.4 ± 1012308.0 (↓) ***	Aminobenzoate degradation
D-Alanyl-D-serine	C_6_H_12_N_2_O_4_	C19719	12043348.0 ± 2309608.7 (↑) **	7399526.5 ± 2020391.2	13778172.7 ± 1928671.1 (↑) ***	Vancomycin resistance
4-(2-Aminophenyl)-2,4-dioxobutanoic acid	C_10_H_9_NO_4_	C01252	2198559.1 ± 929802.0 (↓)	21286247.2 ± 14321200.0	5825576.4 ± 2523295.2 (↓)	Trp metabolism
4,5-Dihydroorotic acid	C_5_H_6_N_2_O_4_	C00337	25364623.6 ± 6702123.4 (↓) ***	52265843.1 ± 10101104.5	40703852.0 ± 3844747.1 (↓) *	Pyrimidine metabolism, biosynthesis of cofactors
(S)-beta-Tyrosine	C_9_H_11_NO_3_	C21308	3274946.3 ± 541643.5 (↑) **	1586651.5 ± 444567.2	2661483.0 ± 937355.2 (↑) *	Biosynthesis of enediyne antibiotics, biosynthesis of secondary metabolites
Indole-3-carboxylic acid	C_9_H_7_NO_2_	C19837	12440031.0 ± 4077586.6 (↑)	4256791.3 ± 1992599.9	20454116.8 ± 10755099.5 (↑) **	Trp metabolism

*P < 0.05, **P<0.01, ***P<0.001 vs. DSS group.

↑ and ↓ represent increases and decreases vs the DSS group, respectively.

### The Influence of Rg1 on Serum Tryptophan Metabolism

**Figure 6:**
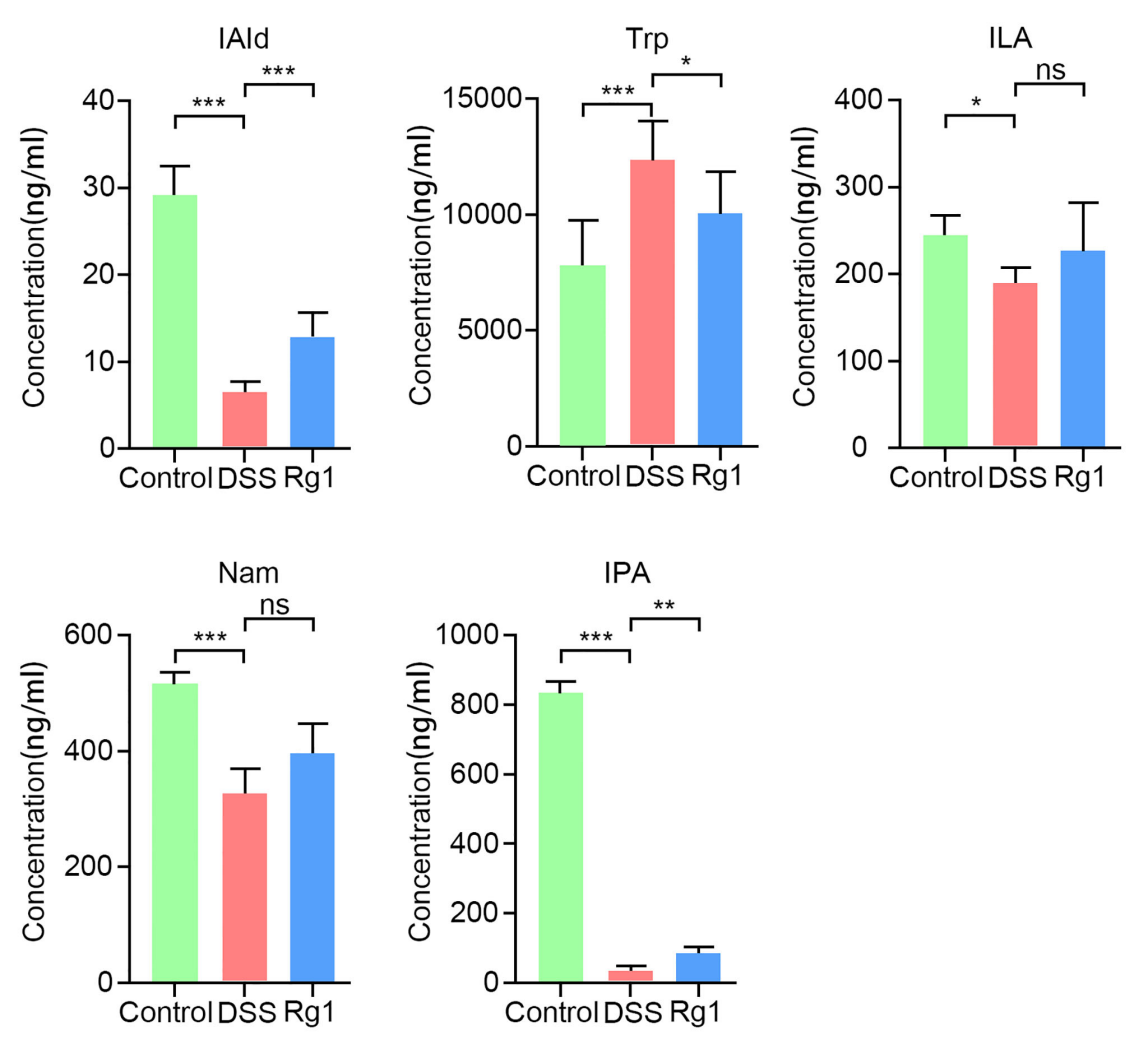
The change of serum tryptophan and its metabolites in all groups. Data are shown as mean ± SD (n=6). *P* < 0.05 was considered to be statistically significant. Significance levels are indicated as *P < 0.05, **P < 0.01, ***P < 0.001, and ns stands for not statistically significant.

The results of global profiling of fecal samples have disclosed that the most prominent metabolic alteration was microbial Trp metabolism which has been reported to be closely related to the integrity of intestinal barrier function. However, since the process of metabolites entering the systemic circulation is regulated by many factors, the content of metabolites in fecal samples does not mean that the same trend can be also observed in serum. Therefore, determining whether the level of Trp and its metabolites in serum is effectively changed by DSS and Rg1 is critical for explaining the mechanism of Rg1 in the treatment of UC. The serum level of Trp and its metabolites was further quantified by targeted analysis. Results of targeted data suggested that Trp metabolism was dynamically changed by DSS and Rg1. As shown in **Figure 6**, the level of Trp and its metabolites, including IAld (*P* < 0.001), ILA (*P* < 0.01), IPA (*P* < 0.001), and Nam (*P* < 0.001), was significantly reduced in DSS group compared with the control group. However, the levels of IAld (*P* < 0.001), ILA, and IPA (*P* < 0.01) were increased after Rg1 treatment. In addition, compared with the DSS group, the concentration of serum Trp is lower in the control and Rg1 groups.

### Correlation Analysis Among Phenotype, Gut Microbiota, and Tryptophan and Its Metabolites

Pearson correlation analysis was adopted to assess the potential connection among phenotype, the altered gut microbiota, and Trp and its metabolites. Between phenotype and gut microbiota level as shown in **Figure S4A**, fecal viscosity and rectal bleeding were significantly positively correlated with *Odoribacter* (r= 1.000, *P* < 0.05; r= 1.000, *P* < 0.05) and were significantly negatively correlated with *norank_f_Muribaculaceae* (r= -0.999, *P* < 0.05; r= -0.999, *P* < 0.05) and *Lactobacillus* (r= -0.998, *P* < 0.05; r= -0.998, *P* < 0.05). Body weight was significantly positively correlated with *Akkermansia* (r= 1.000, *P* < 0.05) and *Verrucomicrobia* (r= 1.000, *P* < 0.05). Colon length was significantly positively correlated with *Lactobacillus* (r= 0.998, *P* < 0.05), *Verrucomicrobia* (r= 1.000, *P* < 0.01), and *Akkermansia* (r= 1.000, *P* < 0.01). Between phenotype and Trp metabolism level as shown in **Figure S4B**, fecal viscosity and rectal bleeding were significantly negatively correlated with Nam (r= -1.000, *P* < 0.05; r= -1.000, *P* < 0.05) and IAld (r= -1.000, *P* < 0.05; r= -1.000, *P* < 0.05). Body weight was significantly positively correlated with IPA (r = 0.997, *P* < 0.05). In addition, as shown in **Figure S4C**, the correlation analysis results between gut microbiota and Trp metabolism demonstrated that Nam and IAld were significantly positively correlated with *norank_f_Muribaculaceae* (r= 1.000, *P* < 0.05, r= 1.000, *P* < 0.05) and were significantly negatively correlated with *Odoribacter* (r= -0.999, *P* < 0.05, r= -0.999, *P* < 0.05). IPA was significantly positively correlated with *Allobaculum* (r=1.000, *P* < 0.01).

## Discussion

Inflammatory bowel disease refers to a series of immune-mediated inflammatory diseases of the gastrointestinal tract. UC is one of several classic inflammatory gastrointestinal diseases (2). The main pathological changes of UC patients are inflammatory lesions of the colon and rectal submucosa, and its main clinical symptoms are weight loss, blood in the stool, diarrhea, etc. (3). In the study, 2.5% DSS-induced mice colitis model is consistent with the pathological changes and clinical symptoms of UC patients. The phenomenon suggested that we have successfully established an animal UC model and relieving UC by drugs in this animal model has certain clinical reference significance. Although 2 mg/10g b.w. Rg1 treatment did not significantly improve the clinical manifestations of UC, such as weight loss, bloody stool, and diarrhea, Rg1 has shown an obvious protective effect on the intestinal barrier and anti-inflammatory effect in UC mice. These findings indicate that Rg1 has a beneficial effect on the treatment of UC and encouraged us to further explore the underlying mechanism by which Rg1 alleviates UC.

Increasing evidence strongly suggests that UC results from an inflammatory response to the abnormal changes in gut microbiota (37). Therefore, to explore the underlying mechanism of Rg1 in the treatment of UC, 16s sequencing technology was adopted to detect the composition of fecal gut microbiota in mice. A growing body of research has confirmed that the richness of gut microbiota will be decreased in DSS-induced UC mice (38), and the same result was shown by alpha diversity analysis in our study. However, Rg1 treatment increased the decreased richness of gut microbiota caused by the DSS challenge. In addition, PCoA and hierarchical clustering analysis showed that the three groups are significantly separated. At the phylum level, the challenge of DSS can induce the abnormal change in gut microbiota, such as dramatically increasing the relative abundance of *Proteobacteria* and dramatically decreasing the relative abundance of *Verrucomicrobia* compared with the control group, and the change caused by DSS can be relieved *via* Rg1 treatment. These results indicate that the DSS challenge and Rg1 treatment significantly changed the overall structure of gut microbiota.

At the genus level, DSS can reduce the relative abundance of potentially beneficial bacteria and the consumption of Rg1 can partly counteract the effect of DSS. Studies have reported that *norank_f*_*Muribaculaceae* may be a bacterium enriched in the healthy intestinal environment and may be potentially beneficial for relieving inflammation (33, 39, 40). In our study, DSS can significantly reduce the relative abundance of *norank_f_Muribaculaceae*, while the relative abundance of *norank_f_Muribaculaceae* was increased after Rg1 treatment compared with the DSS group. Correlation analysis results displayed that *norank_f*_*Muribaculaceae* was negatively correlated with fecal viscosity and rectal bleeding. As a type of bacteria that settles on the mucus layer and uses mucin as a source of nutrition, the relative abundance of *Akkermansia* has been reported to be negatively correlated with intestinal inflammation and the *Akkermansia* species can enhance the function of the intestinal barrier by promoting tight junctions in intestinal epithelial cells (41, 42). DSS can inhibit the level of *Akkermansia* and Rg1 can partly cut down the effect of DSS on *Akkermansia.* Correlation analysis results disclosed that *Akkermansia* was significantly positively correlated with body weight. *Lactobacillus*, known as an important probiotic, exerts several potentially beneficial roles including immune stimulation, pathogen exclusion, and general intestinal health (43). More and more research has disclosed that *Lactobacillus* has a protective effect on UC (44, 45). In our study, DSS can induce the decrease of *Lactobacillus* and the decreased relative abundance of *Lactobacillus* was increased by Rg1 treatment. Correlation analysis demonstrated that *Lactobacillus* was significantly positively correlated with colon length and was significantly negatively correlated with fecal viscosity and rectal bleeding. These results indicate that Rg1 can exert an anti-colitis effect by promoting the growth of potentially beneficial bacteria.

In addition to the potentially beneficial bacteria, at the genus level, Rg1 also inhibited the growth of conditional pathogens which was promoted by DSS. Research has reported that *Odoribacter* was regarded as a short-chain fatty acid-producing bacteria (46, 47). However, many researchers showed that the increased relative abundance of *Odoribacter* has been found in UC mice and patients, and *Odoribacter* was regarded as opportunistic pathogens (48, 49). In our study, DSS significantly elevated the relative abundance of *Odoribacter* compared with the control group, and the level of *Odoribacter* was reduced by Rg1 treatment. It is speculated that the relative abundance of *Odoribacter* may be positively correlated with the occurrence of UC and the correlation analysis confirmed that *Odoribacter* was significantly positively correlated with fecal viscosity and rectal bleeding. *Turicibacter* has been reported to promote the release of inflammatory cytokines (50, 51). In our study, *Turicibacter* was not detected in the control group and DSS group, however, a high relative abundance of *Turicibacter* was detected in the DSS group. For *Bacteroides*, although reports have suggested that the level of *Bacteroides* is lower in UC patients compared with normal individuals and capsular polysaccharide from *Bacteroides fragilis* have a protective effect on the UC (52, 53). However, many studies also have demonstrated that an abundance of sequences from *Bacteroides* in the mucosal tissue of patients with UC compared with normal individuals and *Bacteroides fragilis* maybe induce UC by stimulating the high expression of host IL-17 and enhancing the permeability of intestine epithelial cells (54, 55). Compared with the control group, the relative abundance of *Bacteroides* was significantly increased in our study, and treatment of Rg1 effectively reverses the increase of *Bacteroides*. These results suggest that the suppression effect of Rg1 on the growth of potentially pathogenic bacteria may be the mechanism of Rg1 in the treatment of UC.

The gut microbiota metabolites, such as short-chain fatty acids, bile acids, and Trp and its metabolites, play an indispensable role in protecting the intestinal barrier and regulating immune homeostasis (56–58). For instance, short-chain fatty acids are essential for gut integrity by regulating the luminal pH, influencing mucus production, providing fuel for epithelial cells, and regulating intestine inflammatory (59, 60). Intestinal microbiota metabolites of Trp (ILA, IPA, IAld) both AHR agonists can result in the reduction of IFN-*γ*, IL-6, IL-12, TNF-*α*, IL-7, and IL-17 during inflammatory conditions (61–63). In UC patients and animals, the disturbance of gut microbiota metabolism also has been reported (24, 64, 65). To identify whether DSS can induce the disturbance of microbial metabolism and mechanism of Rg1 in the treatment of UC is related to the regulation of Rg1 on microbial metabolism, we screened the global metabolites profiling of fecal samples. The PCA score plots, OPLS-DA score plots, and volcano map disclosed that DSS and Rg1 induce the change in microbial metabolism among the three groups. In addition, we screened different metabolites among three groups and found 13 metabolites with a dose-effect relationship. In the metabolic pathways related to these metabolites, the most prominent metabolism pathway alteration is the Trp metabolism pathway. Therefore, we speculate that the change of microbial metabolism caused by DSS and Rg1 may be responsible for the occurrence and treatment of UC, respectively. In these microbial metabolic pathways, the microbial metabolism of Trp may be the pathway with the greatest importance.

Global metabolites profiling has suggested that Rg1 and DSS influence the gut microbiota metabolism and microbial metabolism of Trp is the most important metabolic pathway, but the result is that only the metabolic pathways and metabolites that may be regulated have been screened out. Deeper and more precise research needs to be carried out to strengthen the reliability of the results and the depth of research. Given the importance of Trp and its metabolites on intestinal homeostasis and the global metabolites profiling results, the level of Trp and its metabolites in the serum was analyzed. In the serum, microbial metabolites of Trp including IAld, IPA, ILA, and Nam were significantly reduced by DSS. Compared with the DSS group, IAld, IPA, ILA, and Nam were significantly increased after Rg1 treatment. IAld, IPA, and ILA have been reported to be ligands of AHR. AHR is a transcription factor ubiquitously expressed in immune and non-immune cells of the gut (64). Activation of AHR has been reported to ease inflammation and enhance intestinal barrier function in various ways such as reducing the release of pro-inflammatory factors, up-regulating the production of IL-22 which is a cytokine that can exert either inflammatory or protective effects, and inhibiting the increase in MLCK expression and MLC phosphorylation which induced by TNF-*α*/IFN-*γ* (12, 62, 66–68). In addition, a higher level of Trp was detected in the DSS group compared with the control and Rg1 groups. These results suggest that the DSS challenge significantly changed Trp metabolism and Rg1 can effectively cut down the effect of DSS on the Trp metabolism. In addition, the influence of DSS challenge and Rg1 treatment on Trp metabolism may be conducive to the occurrence and mitigation of UC, respectively.

The metabolic pathway of Trp has been reported to have the following three pathways: the kynurenine pathway, the serotonin pathway, and the microbiota metabolic pathway (29). The gut microbiota is mainly involved in the microbiota metabolic pathway. Many researchers have disclosed that *norank_f_Muribaculaceae*, *Lactobacillus*, *Allobaculum*, and *Akkermansia* can catabolize Trp to Trp metabolites with AHR agonistic activity (69–74). In the study, the relative abundance of *norank_f_Muribaculaceae*, *Lactobacillus*, *Allobaculum*, and *Akkermansia* and Trp metabolites (IAld, ILA, IPA, and Nam) was decreased in the DSS group compared with the Control group. Rg1 treatment can increase the relative abundance of these microorganisms and the level of Trp metabolites. In addition, correlation analysis discovered that Nam and IAld were positively correlated with *norank_f_Muribaculaceae*. In the study, the change of the relative abundance of *Allobaculum* is in line with the change of plasm IPA. Correlation analysis also showed that IPA was significantly positively correlated with *Allobaculum*. The results indicate that *Allobaculum* may play an important role in the conversion of Trp into IPA. Based on the above discussion, we can infer that Rg1 may regulate the microbial Trp metabolism by directly regulating these microorganisms.

## Conclusions

In our study, we examined the anti-colitis activity of Rg1 in the UC model induced by DSS and want to explore the underlying treatment mechanism. Oral consumption of 2 mg/10g b.w. Rg1 effectively protected the intestinal barrier and reduced inflammation in UC mice. DSS challenge caused disorders of the overall structure of gut microbiota, promoted the growth of potentially pathogenic bacteria, and inhibited the growth of beneficial bacteria. However, the treatment of Rg1 can effectively alleviate the effects of DSS on the gut microbiota composition of UC mice. DSS and Rg1 can lead to a significant change in gut microbiota metabolism and the most prominent metabolic alteration was Trp metabolism of gut microbiota. In UC mice, the conversion of Trp into Trp microbial metabolites was suppressed and Rg1 treatment can promote the conversion. The mechanism of Rg1 in regulating Trp metabolism is related to the modulative effects of Rg1 on gut microbiota. In summary, Rg1 can protect the intestinal barrier and reduce inflammation of UC mice potentially by regulating gut microbiota and microbial Trp metabolism.

## Data Availability Statement

The datasets presented in this study can be found in online repositories. The names of the repository/repositories and accession number(s) can be found below: NCBI BioProject database, accession number PRJNA814730.

## Ethics Statement

The animal study was reviewed and approved by Animal Ethics Committee in Chengdu University of Traditional Chinese Medicine.

## Author Contributions

WF and CP conceived and proposed the idea; HC, JL, DZ, and JW performed the experiments; HC, JL, and DZ wrote and revised the manuscript; JL, YT, and CP checked the manuscript. All authors have read and approved the final manuscript.

## Funding

This work was supported by the National Natural Science Foundation of China (No. 82104409, 81891012, 81891010, U19A2010, China), China Postdoctoral Science Foundation (No. 2021M690490, China), Sichuan Science and Technology Program (No. 2021YJ0466, China), and the “Xinglin Scholar” Plan of Chengdu University of Traditional Chinese Medicine (No. BSH2020017, China).

## Conflict of Interest

The authors declare that the research was conducted in the absence of any commercial or financial relationships that could be construed as a potential conflict of interest.

## Publisher’s Note

All claims expressed in this article are solely those of the authors and do not necessarily represent those of their affiliated organizations, or those of the publisher, the editors and the reviewers. Any product that may be evaluated in this article, or claim that may be made by its manufacturer, is not guaranteed or endorsed by the publisher.
