# Supplementary material for: Ginsenoside Rg1 Alleviates Acute Ulcerative Colitis by Modulating Gut Microbiota and Microbial Tryptophan Metabolism
Source: Front Immunol. 2022 May 17;13:817600. doi: 10.3389/fimmu.2022.817600 (PMC9152015; doi:10.3389/fimmu.2022.817600)
Supplement: Supplementary file 1 [file DataSheet_1.docx]

**Ginsenoside Rg1 alleviates ulcerative colitis by modulating gut microbiota and microbial tryptophan metabolism**

**Hao Cheng^1#^, Juan Liu^1#^, Dandan Zhang^1^, Jing Wang^1^, Yuzhu Tan^1,2^, Wuwen Feng^1,2*^, Cheng Peng^1,2*^**

^1^State Key Laboratory of Southwestern Chinese Medicine Resources, School of Pharmacy, Chengdu University of Traditional Chinese Medicine, Chengdu, China

^2^The Ministry of Education Key Laboratory of Standardization of Chinese Herbal Medicine, School of Pharmacy, Chengdu University of Traditional Chinese Medicine, Chengdu, China

^#^These authors have the equal contribution to this work.

*** Correspondence:**Wu-wen Feng, email: jiaoxiake-1@foxmail.com; Cheng Peng, email: pengchengcxy@126.co

**TABLE 1S** | Standard curves and other relevant information of tryptophan and its derivatives

| Substance name | Keep time | Linear equation | Correlation coefficient | Quantitative range | Limit of quantitation |
| --- | --- | --- | --- | --- | --- |
| Indole-3-carboxaldehyde (IAld) | 8.84 | y=0.795x+0.109 | 0.9929 | 0.0244-25 | 0.0244 |
| 3-Indolepropionic acid (IPA) | 10.12 | y=0.376x+0.0206 | 0.9994 | 0.195-50 | 0.195 |
| Indole-3-lactic acid (ILA) | 8.50 | y=0.0331x-0.0163 | 0.9966 | 0.488-250 | 0.488 |
| Nicotinamide (Nam) | 3.16 | y=0.203x+0.0203 | 0.9981 | 0.122-125 | 0.122 |
| L-Tryptophan (Trp) | 5.43 | y=0.227x+0.0319 | 0.9908 | 0.122-250 | 0.122 |

**TABLE 2S** | Ion pair information for mass spectrometric detection of tryptophan and its derivatives

| Compounds | Q1 | Q3 | DP | EP | CE | CXP |
| --- | --- | --- | --- | --- | --- | --- |
| Indole-3-carboxaldehyde (IAld) | 146.120 | 117.900 | 71 | 10 | 21 | 10 |
| 3-Indolepropionic acid (IPA) | 189.993 | 130.100 | 66 | 10 | 19 | 12 |
| Indole-3-lactic acid (ILA) | 206.081 | 118.000 | 71 | 10 | 31 | 10 |
| Nicotinamide (Nam) | 123.057 | 80.000 | 76 | 10 | 29 | 6 |
| *L*-Tryptophan (Trp) | 205.037 | 187.900 | 41 | 10 | 15 | 18 |


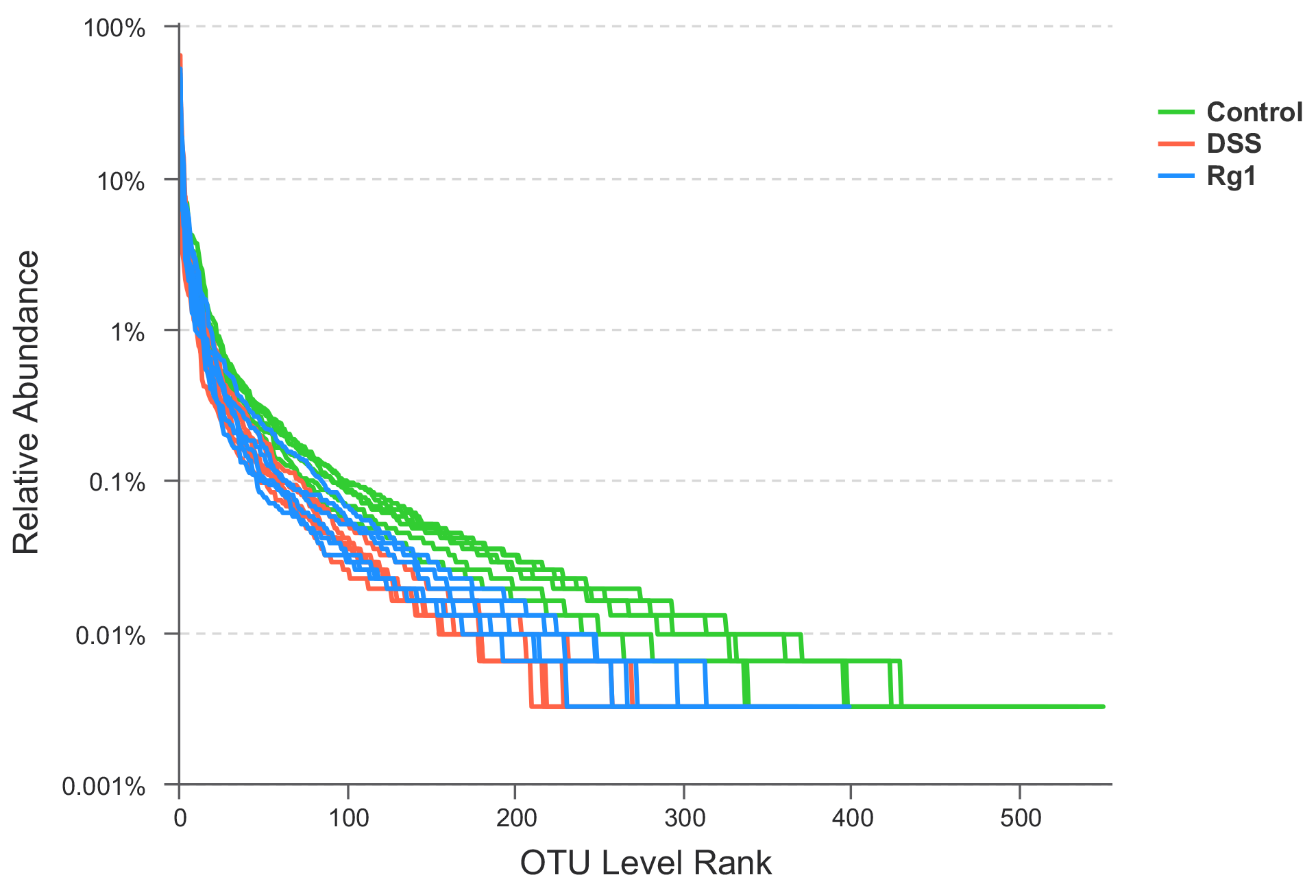


**FIGURE 1S** | The OTU dilution curve of gut microbiota.


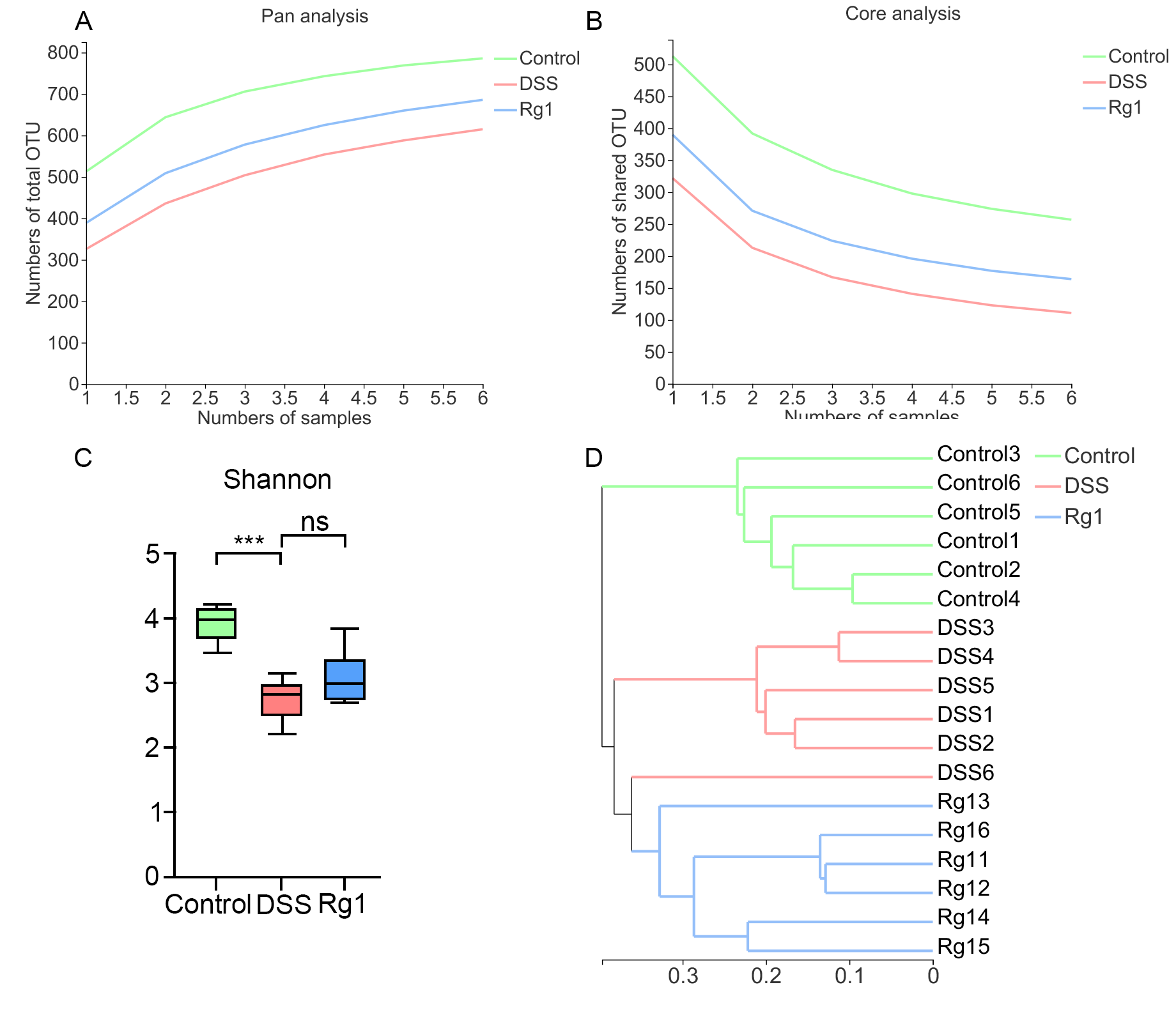


**FIGURE 2S** | The change of gut microbiota after DSS and Rg1 intervention. **(A)** Pan analysis based on OTU level. **(B)** Core analysis based on the OUT level. (**C**) Shannon index shown the change of gut microbiota community richness. **(F)** Hierarchical clustering shows the aggregation of samples in each group.

**
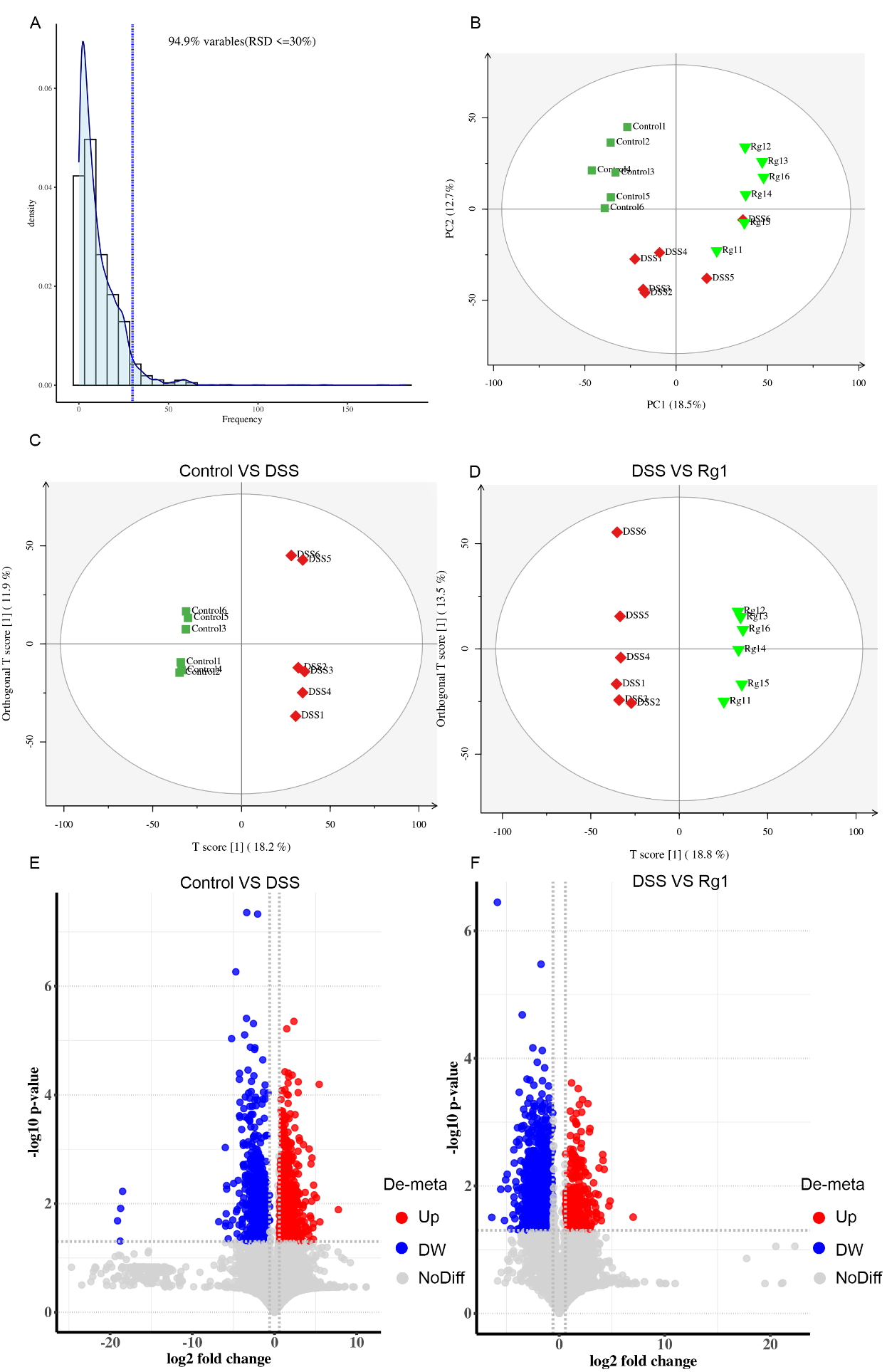
**

**FIGURE 3S** | Rg1 modulated the fecal metabolism in ESI negative ion mode. **(A)** QA analysis result shows the reliability of the data. **(B)** PCA sore plots shows that reflects the distribution of all samples. **(C)** OPLS-DA sore plot reflects the difference between the Control group and the DSS group. **(D)** OPLS-DA sore plot reflects the difference between the DSS group and Rg1 group. **(E)** Volcano map reflects the different specific metabolites between Control group and DSS group. **(F)** Volcano map reflects the different specific metabolites between DSS group and Rg1 group.


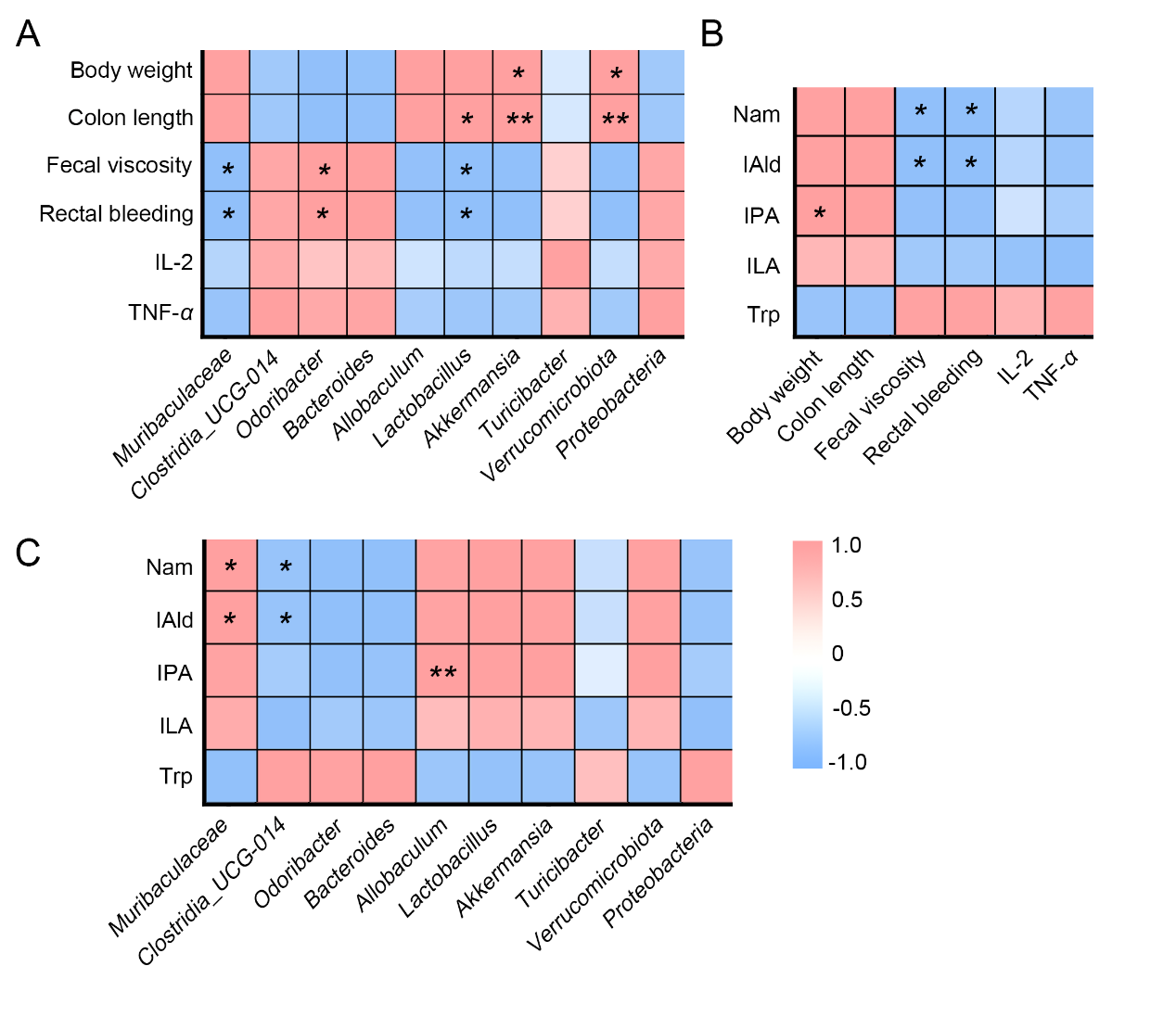


**FIGURE 4S** | Pearson correlation plots of three different levels. **(A)** Correlation analysis results between gut microbiota and phenotype. **(B)** Correlation analysis results between tryptophan and its derivatives and phenotype. **(C)** Correlation analysis results between gut microbiota and tryptophan and its derivatives. *P* < 0.05 was considered to be statistically significant. Significance levels are indicated as **P* < 0.05, ***P* < 0.01, and ****P* < 0.001.
